# From Variant Interpretation to Biomarker Translation: Multi‐omics Integration in Inherited Neuromuscular Diseases

**DOI:** 10.1155/humu/9236120

**Published:** 2026-07-21

**Authors:** Suming Zhang, Xiaoling Lang, Lunxin Liu

**Affiliations:** ^1^ Department of Radiology, Key Laboratory of Obstetric & Gynecologic and Pediatric Disease and Birth Defects of Ministry of Education, West China Second University Hospital, Sichuan University, Chengdu, Sichuan, China, scu.edu.cn; ^2^ Office of Operations Management, West China Second University Hospital, Sichuan University, Chengdu, Sichuan, China, scu.edu.cn; ^3^ Department of Neurosurgery, West China Hospital, Sichuan University, Chengdu, Sichuan, China, scu.edu.cn

**Keywords:** artificial intelligence, biomarkers, clinical translation, genetic neuromuscular diseases, multi-omics integration, variant of uncertain significance

## Abstract

Genetic neuromuscular diseases are highly heterogeneous disorders characterized by diagnostic challenges and limited therapeutic options, underscoring an urgent need for precise biomarkers. The rapid advancement of multi‐omics technologies has broadened biomarker discovery from single genomics to multidimensional integrative analyses encompassing transcriptomics, proteomics, and metabolomics. This progression offers opportunities to improve disease diagnosis, subtyping, prognosis assessment, and treatment monitoring. However, translational gaps persist between multi‐omics discoveries and clinically applicable biomarkers. This review systematically examines the current application of multi‐omics biomarkers in genetic neuromuscular diseases. It provides an in‐depth analysis of the multifaceted barriers encountered during the translation process, including technical hurdles, clinical validation complexities, data interpretation challenges, and health system‐level obstacles. Furthermore, the review explores emerging solutions including artificial intelligence‐assisted decision‐making, ethical governance, and policy preparedness. The review aims to offer a framework for constructing a potentially responsible and efficient multi‐omics translation in genetic neuromuscular diseases.

## 1. Introduction

Genetic neuromuscular diseases (NMDs) comprise a broad and heterogeneous group of disorders involving the lower motor neurons, peripheral nerves, neuromuscular junction, and skeletal muscle, many of which arise from pathogenic genetic variation that disrupts neuromuscular development, maintenance, or function [1]. This spectrum includes monogenic or predominantly mutation‐driven conditions such as spinal muscular atrophy (SMA), Duchenne muscular dystrophy (DMD), Charcot–Marie–Tooth (CMT) disease, limb‐girdle muscular dystrophy (LGMD), and mitochondrial myopathy, as well as genetically heterogeneous or molecularly informed disorders such as amyotrophic lateral sclerosis (ALS) and myasthenia gravis (MG) [2]. Although advances in genomic medicine have substantially improved the identification of disease‐causing variants, the marked clinical heterogeneity, variable disease trajectories, and complex treatment responses across neuromuscular disorders remain unchanged [3]. This translational gap has sparked growing interest in multi‐omics approaches that may help bridge variant‐level discovery with downstream molecular pathology, clinically meaningful stratification, and biomarker development.

The advent and integration of multi‐omics technologies, spanning genomics, transcriptomics, proteomics, metabolomics, and epigenomics, offer a potential pathway to address these longstanding challenges in genetic NMDs [1, 4]. By providing a holistic, systems‐level view of molecular interactions, multi‐omics approaches go beyond single‐variant identification. They can enhance precision diagnosis, characterize phenotypic expression, and improve disease classification [5]. Several cohort studies in NMD have shown that the integration of whole‐genome sequencing and transcriptome RNA‐seq can further increase the rate of diagnosis by 30%–40% in patients who remain undiagnosed after exome sequencing [6–8]. Thus, multi‐omics integration is synergistic rather than merely additive. It can reveal molecular signatures of disease subtypes and individual patients, supporting precision medicine in this challenging field [9].

The primary impetus for adopting multi‐omics strategies in genetic NMDs is the compelling opportunity to discover and validate robust biomarkers that can bridge the gap between molecular pathology and clinical utility. Current biomarker strategies in NMDs, although informative, often have limited sensitivity, specificity, and interpretation of disease activity [10]. Recently, the shift toward multi‐omics has been driven by the critical goal of translating molecular complexity into actionable clinical tools, including biomarkers that can reduce diagnostic delays, predict therapy response, support disease stratification, and ultimately improve outcomes for patients with rare and heterogeneous neuromuscular disorders [11]. Therefore, this review aims to examine the current translational landscape of multi‐omics biomarkers in genetic NMDs, with a focus on their diagnostic, stratification, prognostic, and treatment‐monitoring applications, the shared barriers that limit clinical implementation and the policy, governance, and interpretive frameworks required for responsible translation.

## 2. Current Status of Multi‐omics Biomarker Research Across Translational Use Cases

To clarify where multi‐omics biomarkers are currently contributing most and where translational maturity remains limited, the current literature were organized according to four major clinical use cases: diagnostic biomarkers, treatment‐response and pharmacodynamic (PD) biomarkers, disease‐subtyping and progression‐stratification biomarkers, and prognostic or progression‐risk biomarkers (Table 1).

**Table 1:** Evidence‐oriented classification of multi‐omics biomarkers in inherited neuromuscular diseases.

Biomarker type	Specific biomarker	Sample size/evidence source	Omics or measurement method	Limitations
Diagnostic biomarker	Causal or likely causal variants in recurrent NMD genes, especially DMD, TTN, NEB, RYR1, ACTA1, and MTM1, supported when needed by aberrant RNA or protein evidence	247 families with suspected monogenic NMDs [7]	ES, RNA‐seq	Variant interpretation pipelines still limit routine reproducibility.
Diagnostic biomarker	DMD structural variants, DMD splice‐altering variants, and second‐hit variants in recessive NMD genes revealed after exome‐negative or single‐hit testing	247‐family cohort [7]	Muscle RNA‐seq	Partly phenotype‐driven.
Diagnostic biomarker	CAPN3 full‐length transcript and calpain‐3 protein in muscle, fibroblast, or urine	60 LGMD patients [12]	Targeted DNA sequencing	Limited validation cases. External replication and standardized thresholds are needed.
Treatment‐response biomarker	Full‐length SMN2 mRNA/truncated SMN2 transcript ratio	Review‐level synthesis of SMA treatment‐era studies and trials [13]	Transcriptomics	Clinical thresholds are not stable across sample types, ages, treatment timing, or platforms.
Treatment‐response biomarker	Neurofilament markers combined with electrophysiology in SMA, including NfL/pNfH and CMAP	45 individuals with SMA in a neonatal‐to‐adult cohort [14]	Fluid protein assays in serum/CSF plus neurophysiology	Small sample size. Neurofilament is not disease‐specific and is influenced by age, treatment status, and active denervation.
Treatment‐response biomarker	Plasma and CSF multi‐omics response markers in SMA, including NEFH, NEFL, IFI30, PMVK, creatinine, and lipid metabolites	53 baseline plasma SMA samples vs 71 plasma controls [15]	Targeted metabolomics, targeted proteomics, Olink neurology panel, electrochemiluminescence assays, LC‐MS/MS	Retrospective design, missing clinical data, small subgroups. Preanalytical variability and imputation may bias results; independent validation is required.
Disease subtyping biomarker	CAPN3 variant class and calpain‐3 deficiency for dominant versus recessive calpainopathy	Four dominant LGMD cases with calpain‐3 deficiency, plus contextual CAPN3 variant evidence [16]	GS, pedigree analysis, Western blotting, structural modeling, phenotype correlation	Mechanistically informative but still variant‐ and family‐specific. Novel variants require segregation, functional validation, and database reclassification over time.
Disease subtyping biomarker	4q35 hypomethylation and structural variant context distinguishing FSHD1 from FSHD2	Genetically FSHD1 subjects [17]	Epigenomics and structural variant analysis, including methylation profiling and D4Z4/4q35 assessment	Assay platforms and interpretation rules differ across centers. Clinical subtype boundaries require harmonized methylation and structural‐variant workflows.
Prognostic biomarker	Early cardiac and autonomic markers in DMD, including HRV, ECG, echocardiographic indices, and LVEF	66 ambulant DMD boys aged 5–10 years; HRV controls *n* = 46 and ECG controls *n* = 31 [12]	Physiological and imaging measures: HRV, ECG, echocardiography; and genotype‐phenotype association	Single‐center and cross‐sectional design. Longitudinal cardiac outcomes are needed to prove patient‐level prognostic value.
Prognostic biomarker	PLS3 and NCALD as SMA disease‐course modifiers	Review‐level synthesis of human and preclinical modifier studies [13]	Genomics/transcriptomics and modifier‐gene expression studies	Translation requires prospective cohorts and integration with SMN2 copy number and clinical trajectory.
Prognostic biomarker	Serum creatinine and related metabolic markers of muscle wasting in SMA	Treatment‐era SMA biomarker review and longitudinal multi‐omics evidence, including the 53‐sample plasma baseline cohort in [15] and review synthesis in [18]	Clinical chemistry plus metabolomics	Creatinine is not a disease‐specific biomarker and may be biased by other factors.

Abbreviations: DMD, Duchenne muscular dystrophy; ECG, electrocardiography; ES, exome sequencing; GS, genome sequencing; HRV, heart‐rate variability; LGMD, limb‐girdle muscular dystrophy; LVEF, left ventricular ejection fraction NMD, neuromuscular disease; SMA, spinal muscular atrophy.

### 2.1. Diagnostic Biomarkers: Improving Molecular Diagnosis, but Not Yet Routine Diagnostic Tools

The most common use of multi‐omics in inherited NMDs is diagnosis, particularly for patients whose cases remain unexplained after exome or genome sequencing. Transcriptomics has been especially valuable in disorders in which the causal lesion is not fully resolved at the DNA level, including DMD, SMA, and large‐gene myopathies such as nebulin‐related disease [14, 19]. A previous study reported that integrating exome sequencing with auxiliary genomic, RNA or protein studies in DMD helped identified splice‐altering and increased the diagnostic rate from 34% to 62% [7]. Similar diagnostic promise has been reported in inherited neuropathies and mitochondrial disorders, where integrated transcriptomic and proteomic readouts can clarify whether a candidate variant produces a measurable functional effect [20–22]. In the diagnostic realm, multi‐omics serve not only to discover new markers, but also to improve the diagnostic interpretation of variants of uncertain significance (VUS) and splice‐altering variants. For instance, transcriptome sequencing (RNA‐seq) has successfully revealed the functional impact of deep intronic variants in COL6A1, demonstrating how they activate cryptic splice sites and create pseudo‐exons that disrupt the reading frame in collagen VI‐related dystrophy [23]. Similarly, in muscular dystrophy, in vitro minigene assays combined with transcriptomics showed that adjacent missense VUSs (c.5628C>A and c.5633A>T) within the DYSF gene adversely affect mRNA splicing, thereby elevating them from ambiguous findings to potential diagnostic biomarkers [24]. By providing functional evidence of aberrant splicing or mapping complex genomic rearrangements, multi‐omics transforms elusive noncoding sequences into potential molecular diagnoses. However, the current diagnostic application is still constrained by several translational weaknesses. Many studies are based on small, highly selected cohorts and analytical platforms that are difficult to standardize across centers [25].

### 2.2. Treatment Response Biomarkers: An Emerging Goal Still Limited by Validation Gaps

A second major application is the development of biomarkers that can monitor therapeutic response. This area has become particularly important in neuromuscular disorders including DMD and SMA, where exon‐skipping therapies, gene‐editing strategies, and splice‐modifying treatments have created an urgent need for treatment‐response and PD markers that go beyond static genotyping [26]. In SMA, tracking the ratio of full‐length *SMN2* mRNA to truncated transcripts in patient biofluids serves as a direct PD biomarker to monitor splicing correction [13]. In DMD, ASO‐mediated exon skipping aims to bypass specific out‐of‐frame deletions; the detection of the skipped mRNA transcript and subsequent quantification of dystrophin protein restoration at the sarcolemma act as primary molecular PD biomarkers [27]. Additionally, tracking circulating surrogate biomarkers, such as phosphorylated neurofilament heavy chain (pNfH) and muscle‐specific microRNAs (myomiRs, e.g., miR‐206), allows clinicians to dynamically monitor axonal rescue and membrane stability in SMA and DMD, translating variant‐level functional correction into measurable systemic readouts [18]. Furthermore, multi‐omics approaches may help interpret complex genotype‐treatment relationships, such as those involving the 1.5 Mb duplication or deletion of the PMP22 gene in Charcot–Marie–Tooth disease Type 1A (CMT1A) and hereditary neuropathy with liability to pressure palsies [18, 28]. Despite intense interest, this remains translationally immature. Many proposed response biomarkers are derived from short‐term or cross‐sectional studies, and few have been prospectively validated against meaningful clinical endpoints, such as sustained functional benefit or treatment‐switching decisions.

### 2.3. Disease Subtyping and Patient Stratification Biomarkers: Biologically Informative, but Not Yet Clinically Stable

Multi‐omics has also been used to identify biologically meaningful subtypes within NMDs, to improve patient stratification for prognosis and treatment selection [15, 29]. A classic example is evaluating SMN2 copy number variants, which provides a critical gene dosage biomarker that robustly stratifies SMA patients into severe infantile‐onset (Type 1) versus milder phenotypes (Types 2 or 3), thereby guiding the timing of genetic interventions [13, 16]. Beyond gene dosage, evaluating specific functional variants in the CAPN3 gene can accurately stratify calpainopathy into the classic autosomal recessive LGMD2A or the milder autosomal dominant LGMD D4, particularly when splice‐altering variants (e.g., c.1490C>T) create alternative transcripts with dominant‐negative effects [30]. Furthermore, epigenetic stratification is pivotal in mapping noncoding structural complexities; for example, epigenetic dysregulation by 4q35 hypomethylation status clearly differentiates facioscapulohumeral muscular dystrophy Type 1 (FSHD1) from FSHD2, demonstrating how integrating epigenetic and structural variant profiles ensures patients are stratified by true biological mechanisms rather than overlapping clinical phenotypes [17]. Yet most stratification biomarkers remain primarily biologically informative rather than clinically actionable. They are often derived from small retrospectively cohorts, and insufficiently linked to prospective outcomes or differential treatment response. As a consequence, current stratification biomarkers are useful for generating mechanistic hypotheses and refining disease models, but not mature enough to guide routine patient allocation or therapy selection.

### 2.4. Prognostic and Progression‐Risk Biomarkers: Strong Clinical Demand, but Limited Patient‐Level Precision

Risk prediction is another area of strong clinical demand, because inherited NMDs often show marked variability in progression, complications, and treatment needs. In DMD, there is intense interest in biomarkers that can predict the development of cardiomyopathy, or the severity of respiratory decline. For instance, autonomic markers have been reported to show potential predictive value for dilated cardiomyopathy [12]. In SMA, multi‐omics studies have identified genetic modifiers such as PLS3 (Plastin‐3) and NCALD that independently influence the disease course. These modifiers act as prognostic biomarkers by affecting axonal F‐actin bundles and endocytic pathways, thereby modulating the severity of disease progression regardless of the primary SMN1 deletion [13]. Additionally, in LGMD2A, an in vitro autolytic function assay measuring the functional magnitude of CAPN3 variants serves as a candidate prognostic biomarker; the loss of autolytic enzymatic activity is highly predictive of rapid disease progression, even in patients where total calpain‐3 protein quantities appear normal on Western blots [31]. Furthermore, longitudinal tracking of surrogate molecular markers, such as serum creatinine (Crn), has shown high predictive power for assessing the deterioration of motor function in SMA, effectively translating variant‐induced muscle wasting into a scalable progression‐risk biomarker [18, 31]. However, many candidate prognostic biomarkers perform reasonably well at the group level but lack sufficient stability, calibration, or incremental value to support individualized risk prediction in practice. Accordingly, prognostic research is advancing, but the field is still far from routinely using multi‐omics biomarkers to make high‐confidence predictions for individual patients.

Taken together, the current literature suggests that multi‐omics biomarker researches in inherited NMDs are most advanced in diagnostic interpretation, increasingly active in treatment‐response monitoring and molecular stratification, and an active area of investigation [32, 33]. Importantly, the gap between discovery and translation is not explained solely by a lack of biological interpretation. Across DMD, SMA, inherited neuropathies, mitochondrial disorders, MG, and even biomarker‐rich comparator fields such as ALS, diverse genetic NMDs share the same dilemma: candidate multi‐omics biomarker are identified, but their movement into clinically actionable tools is slowed by restricted sample access, lack of assay standardization, limited longitudinal validation, uncertain evidence compared with existing common measures, and fragmented clinical workflows [34]. Reorganizing the literature by translational use case, therefore, makes clear that the field does not suffer from a shortage of candidate biomarkers. Rather, it suffers from uneven evidence maturity across the very clinical questions those biomarkers are meant to answer.

## 3. From Discovery to Application: Shared Last‐Mile Barriers

**Figure 1:**
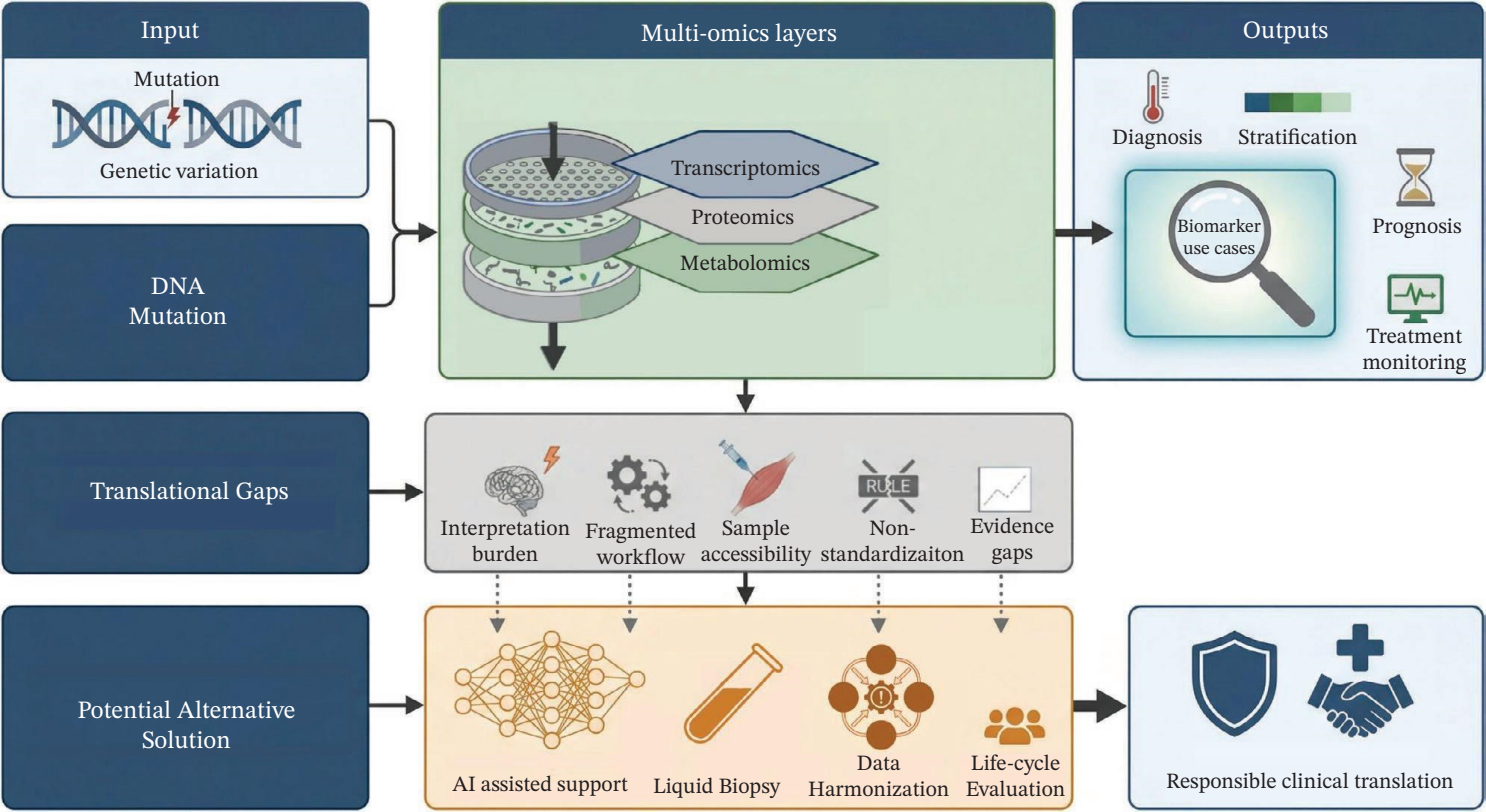
Translational pathway of multi‐omics biomarkers in genetic neuromuscular diseases. Pathogenic genetic variation represents the starting point for biomarker discovery, whereas transcriptomics, proteomics, and metabolomics, provide functional evidence that links DNA‐level variants to downstream molecular alterations. These integrated molecular profiles may support clinically relevant applications, including molecular diagnosis, patient stratification, prognosis, and treatment‐response monitoring. The middle panel summarizes the shared last‐mile barriers that limit clinical translation, including limited sample accessibility, assay variability, evidence gaps and insufficient alignment between preclinical evidence, regulatory standards, and interpretive burden of integrating multi‐omics. The lower panel outlines the implementation‐oriented responses discussed in this review, including AI‐assisted interpretation and evidence synthesis, transparent data governance, clearer reimbursement and regulatory pathways, and workforce readiness for multidisciplinary clinical adoption.

Since the translation of multi‐omics biomarkers is shaped not only by scientific validity, but also by workflow, implementation, and governance. The following sections consider these shared last‐mile barriers through the perspectives of three stakeholder groups closely involved in moving such technologies from laboratory discovery to clinical use: clinical neurologists, laboratory medicine experts, and policymakers. The major translational gap and potential alternative solution are summarized in Figure 1.

### 3.1. Clinical Translation Is Limited by Interpretation Burden, Workflow Fragmentation, and Reporting Challenges

From the perspective of clinical neurology, the complex interpretation burden of integration multi‐omics data, and the clinical workflows are major barriers of translating to clinical use. In neuromuscular disorders, this challenge is increased by marked genetic heterogeneity, variable tissue involvement, and overlapping clinical phenotypes, all of which complicate distinguishing disease‐relevant signals from secondary molecular changes and incidental findings.

Effective interpretation, therefore, often depends on multidisciplinary collaboration involving clinical geneticists, neurologists, laboratory specialists, and, in some cases, bioinformaticians and radiologists [34]. However, such collaboration is resource‐intensive and not easily standardized across centers [35]. multi‐omics clinical implementation is also slowed by fragmented workflow. The lack of unified gold‐standard workflows for sample processing, and postprocessing data analysis protocols undermines the robustness and reproducibility of multi‐omics in clinical translation [36].

Responsibility for data interpretation, result communication, and downstream clinical action may also remain poorly defined, particularly when multi‐omics findings are suggestive rather than definitive [35, 37]. As a result, the process of moving from high‐dimensional molecular data to a clinically actionable conclusion can be slow, labor‐intensive, and unevenly implemented across institutions. A related challenge lies in report generation and communication. Even when potentially meaningful molecular patterns are identified, translating them into reports that are interpretable, clinically relevant, and usable by both specialists and nonspecialists remains difficult [38]. This is especially important in genetic NMDs, where clinical decision‐making often depends on longitudinal monitoring, careful phenotype interpretation, and coordination across multiple disciplines. AI‐assisted technologies, particularly large language models (LLMs), hold considerable promise for improving multi‐omics data interpretation.

### 3.2. Laboratory Implementation Is Constrained by Sample Accessibility, Assay Standardization, and Cross‐Center Reproducibility

From the perspective of laboratory medicine, major barriers are sample accessibility and assay conditions that are not easily harmonized, reproduced, or scaled across centers and real‐world testing environments. Many NMDs require access to muscle, peripheral nerve, or, less commonly, cerebrospinal fluid. However, muscle biopsy is invasive, burdensome, and often impractical for repeated longitudinal sampling, particularly in pediatric populations [39]. Its feasibility is further constrained by variability in disease stage, selective muscle involvement, and the patchy distribution of degeneration, inflammation, fibrosis, or fatty replacement, all of which may influence molecular readouts and complicate interpretation. These limitations have increased interest in blood‐based, CSF‐based, and other minimally invasive liquid biopsy approaches. Such specimens offer practical advantages for repeated sampling, longitudinal monitoring, and multicenter studies. They may be particularly valuable for tracking treatment response in disorders such as dystrophinopathies or SMA. Nonetheless, circulating biomarkers may be influenced by systemic inflammation, comorbidities, age, physical activity, and treatment exposure, thereby reducing disease specificity [40]. As a result, less invasive sampling strategies improve feasibility but do not fully address the challenge of capturing tissue‐ and cell‐type‐specific pathology in a clinically meaningful way.

Multi‐omics assay standardization presents another obstacle. Multi‐omics studies in genetic NMDs are frequently conducted across different centers using heterogeneous protocols for specimen acquisition, processing, storage, sequencing or mass spectrometry platforms, and downstream analytical pipelines. Even before data generation, preanalytical variation, including biopsy site selection, ischemia time, sample preservation, freeze‐thaw cycles, and differences in nucleic acid or protein extraction, can substantially alter transcriptomic, proteomic, and metabolomic profiles [41]. These issues are especially problematic in NMDs, where disease‐related molecular signals may already vary across muscle groups, disease severity, ambulatory status, and treatment history. Downstream inconsistencies in normalization, feature selection, batch correction, and integration strategies further limit comparability across studies and reduce confidence in the reproducibility of candidate biomarkers [42]. Although harmonization may diminish the bias, it cannot solve the heterogeneity from methodological error [43]. Constructing standardized SOP measurement of multi‐omics across centers may be a potential solution in the future.

Such challenges are amplified in rare disease settings because small sample sizes make studies particularly vulnerable to batch effects, overfitting, and center‐specific biases. When cohorts are limited, technical noise can become difficult to distinguish from true biological heterogeneity, and findings derived from a single platform or institution may fail to replicate in independent datasets [44]. This problem is especially relevant in neuromuscular disorders, where clinical heterogeneity is already substantial and where subgroup analyses are often under powered [44]. Accordingly, progress toward clinically useful multi‐omics biomarkers will depend not only on improved sample accessibility but also on harmonized protocols, reference standards, multicenter validation, and study designs explicitly tailored to the constraints of rare NMD research [45].

### 3.3. Health‐System Adoption Is Constrained by Evidence Misalignment, Reimbursement Uncertainty, and Limited Real‐World Validation

From the perspectives of policymakers, payers, and health technology assessment bodies, a major reason why candidate multi‐omics biomarkers remain translationally immature is that the evidence generated in the lab is often insufficiently aligned with the standards required for reimbursement, regulatory approval, and routine clinical adoption in the real word [46].

Analytical validity and biological plausibility may support publication and early enthusiasm. Still, adoption decisions require a different level of certainty: whether a biomarker adds clinically meaningful information beyond existing pathways, whether that added value is reproducible across settings, and whether its use justifies the costs, workflow changes, and downstream consequences associated with implementation. Recent literature on omics technologies and health technology assessment consistently notes that evidence packages are often fragmented, comparator strategies are suboptimal, and outcome definitions remain heterogeneous, making it difficult for decision‐makers to assess value consistently [47].

The sharing and integration of multi‐omics in NMD can both inform policymaking and minimize duplication of effort and resource waste. In accordance with the goals of accelerating omics research and data sharing of IRDiRC (International Rare Disease Research Consortium), the European Commission funded three flagship projects, including RD‐Connect, NeurOmics, and EURenOmics in 2012 [48]. By generating diverse omics profiles, NeurOmics (http://www.rd-neuromics.eu) and EURenOmics (http://www.eurenomics.eu) facilitate improved diagnostic accuracy and therapeutic strategies in rare renal and neurodevelopmental conditions [49], whereas RD‐Connect (http://www.rd-connect.eu) builds a dedicated infrastructure for streamlined data sharing, systematic integration, and comprehensive analytical processing of these resources [48].

A further unresolved issue is that conventional models such as large RCTs do not easily align with the realities of data‐intensive biomarker technologies in rare genetic NMDs [50]. Policymakers are often asked to make decisions under high uncertainty, particularly when candidate biomarkers are derived from small cohorts, validated in specialized centers, and linked to rapidly evolving computational pipelines [51]. In this context, reliance on conventional randomized evidence alone may be unrealistic, FDA evaluate the multi‐omics finding from small, highly selected patient cohorts and depend on complex computational modeling and analysis pipelines cautiously and critically [52]. Therefore, recent policy‐oriented work emphasizes the need for life‐cycle evaluation strategies that couple early implementation with structured postmarket evidence generation, including real‐world data collection, and stronger links between clinical use and longitudinal outcomes [53]. However, such infrastructures remain underdeveloped in most health systems, especially for rare diseases.

Thus, reimbursement uncertainty reflects broader unresolved questions about fairness and system readiness. Decision‐makers consider not only whether a biomarker works under ideal conditions, but also whether it can be implemented equitably across populations and institutions with different resource levels [54]. Recent work in multi‐omics assessment highlights persistent concerns regarding ancestral under‐representation, inconsistent access to specialist centers, limited interoperability of clinical and molecular data, and the absence of robust frameworks for incorporating patient benefit, diagnostic odyssey reduction, and long‐term societal value into reimbursement decisions [34, 55]. Thus, from a policy perspective, the central barrier is not simply a lack of evidence, but the absence of a mature translational ecosystem capable of supporting proportionate regulation, evidence‐generating reimbursement, and accountable implementation at scale.

## 4. AI‐Assisted Interpretation as an Emerging Enabler, Not a Standalone Solution

Interpretation has become a major bottleneck in translating multi‐omics biomarkers for genetic NMDs. This task is especially challenging in rare neuromuscular disorders, where clinically meaningful evidence is often distributed across multiple sources, including unstructured medical records, electrophysiological reports, descriptions of muscle pathology, genetic databases, and a rapidly expanding body of functional literature [56]. In addition, interpretation frequently requires cross‐modal reasoning, such as linking aberrant splicing signals to variant pathogenicity, or relating protein‐ and metabolite‐level disturbances to disease stage, tissue involvement, or treatment exposure [57]. The volume, heterogeneity, and continual updating of such evidence make consistent synthesis difficult in routine clinical practice, particularly in settings where specialist expertise is limited. In this context, AI‐assisted tools, including LLM‐based systems, may be useful for selected interpretive tasks that are text‐heavy, evidence‐intensive, and require integrating fragmented knowledge sources [58].

### 4.1. Potential Roles of LLM‐Assisted Tools in Phenotype Extraction, Evidence Synthesis, and Report Generation

In workflows of diagnosis and treatment for genetic NMDs, AI‐assisted tools may be valuable at several stages. In the preanalytical stage, LLM may help extract phenotypic information from neurology notes, electromyography reports, and pathology descriptions, and map these findings to structured vocabularies, such as the Human Phenotype Ontology. During interpretation stage, AI‐supported systems may assist in aggregating evidence relevant to VUS or multimodal biomarker signals by linking genomic findings with transcript‐level abnormalities, protein perturbations, and recently published functional studies. Such tools may also support multidisciplinary review by summarizing complex case information, prioritizing potentially relevant lines of evidence, and reducing the burden of manual literature retrieval. In the reporting stage, AI‐assisted drafting may help translate high‐dimensional molecular findings into layered outputs tailored to different users, including specialist‐facing summaries for multidisciplinary teams (MDTs) and more accessible explanations for patients and families. These potential roles are best understood as workflow‐support functions rather than as autonomous forms of clinical reasoning.

### 4.2. Human‐AI Teaming in Rare Disease MDT: Opportunities and Limits

For rare NMDs, AI should support but not replace healthcare professionals, with experts retaining responsibility for final diagnosis, prognosis and management decisions [59–61]. Multi‐omics findings often require contextual judgment that depends on phenotype quality, disease trajectory, tissue specificity, and the broader clinical picture, all of which remain difficult to formalize fully. Therefore, clinical geneticists, neurologists, laboratory specialists, and other domain experts remain essential for evaluating the relevance, reliability, and actionability of AI‐supported outputs [62]. In this setting, AI may help reduce the interpretive burden by assisting with information extraction, evidence organization, and draft synthesis, whereas final judgment, communicating uncertainty, and downstream clinical decision‐making remain human responsibilities [63–66]. A team‐based model is also more consistent with current concerns regarding explainability, accountability, and patient safety, particularly in rare disease contexts where evidence is sparse and misclassification may have substantial consequences.

### 4.3. What AI Cannot Solve: Biological, Infrastructural, and Evidentiary Barriers

At the same time, AI should not be framed as a solution to the major structural barriers that continue to limit biomarker translation in rare NMDs. AI cannot compensate for poor‐quality or biologically uninformative samples, nor can it substitute for assay harmonization, cross‐center standardization, or rigorous analytical validation [67–69]. It cannot generate clinical utility evidence when longitudinal and real‐world outcome data are lacking, and it does not automatically address the cohort biases common in rare disease research, including limited ancestral diversity and uneven representation across healthcare settings. More broadly, AI may help mitigate selected interpretive burdens, but it cannot replace the biological, evidentiary, and organizational foundations required for responsible clinical translation. Its role should therefore be understood as augmentative and conditional, rather than transformative in itself.

## 5. Toward Responsible Translation: Policy and Health‐System Readiness

### 5.1. Evidence Standards for Multi‐omics Biomarkers in Rare Diseases

**Figure 2:**
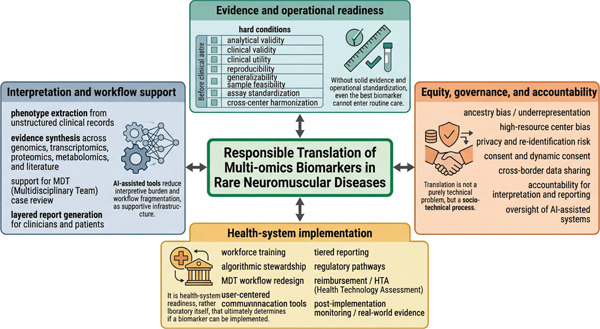
Translational readiness framework for multi‐omics biomarkers in rare neuromuscular diseases. This schematic outlines the main domains that shape whether a candidate biomarker is ready for clinical implementation. Evidentiary maturity refers to analytical validity, biological plausibility, reproducibility across cohorts, and demonstrated incremental clinical utility beyond existing diagnostic or monitoring pathways. Operational feasibility captures whether the required samples, sequencing or mass spectrometry platforms, bioinformatic pipelines, turnaround time, and quality‐control procedures can be standardized across centers. Interpretation and workflow support indicates the need for structured reporting, multidisciplinary review, and decision‐support tools that can translate high‐dimensional molecular signals into clinically actionable conclusions. Equity and governance highlight the importance of representative reference datasets, privacy protection, transparent data sharing, and accountable oversight of AI‐assisted interpretation. Health‐system readiness refers to reimbursement pathways, regulatory alignment, workforce capacity, and real‐world evidence generation. Together, these domains emphasize that biomarker translation depends not only on molecular performance but also on reproducibility, interpretability, implementation capacity, equitable access, and accountable clinical use.

The translation of multi‐omics biomarkers from discovery cohorts to clinical application requires an explicit and fit‐for‐purpose evidentiary framework (Figure 2). In rare NMDs, where biomarker candidates may reflect dynamic processes such as aberrant splicing, protein network disruption, inflammation, fibrosis, or metabolic stress, evidence standards must extend beyond pathogenicity classification alone and address how such signals are measured, interpreted, and used in practice [70]. Variant interpretation guidelines (e.g., ACMG) remain central but do not yet fully cover proteomic and other omics readouts. Thus, new standards are being developed to incorporate quantitative protein abnormalities into pathogenicity assessment [71]. In neuromuscular diseases, incorporating transcriptomic and proteomic biomarkers into diagnostic pipelines is explicitly framed as part of a translational program, where functional, multilayer evidence is combined with clinical context to reach actionable conclusions [5]. More explicit standards of this kind would help distinguish biologically interesting signals from biomarkers that are sufficiently mature for implementation‐oriented assessment.

### 5.2. Equity, Governance, and Accountability in Data and AI‐Mediated Translation

Equity concerns in data and AI‐mediated multi‐omics translation are not external to biomarker development. Still, they are embedded in the reference datasets, literature bases, and computational infrastructures on which interpretation depends. In NMDs, about 86% of published genomic studies are from European‐ancestry populations, and non‐European groups are underrepresented in control databases, directly hampering accurate variant interpretation and genetic diagnosis in all settings [72, 73]. The inequities may be further amplified when LLM‐assisted interpretation tools are trained primarily on English‐language literature and Euro‐American databases, thereby privileging Western evidence ecosystems while under‐weighting locally generated data, non‐English case series, and region‐specific phenotypic patterns [74]. In this sense, equity concerns in data and AI‐mediated multi‐omics translation are intrinsic to current reference datasets and infrastructures.

Growing use of AI and LLMs in genomics and multi‐omics raises linked concerns about explainability, accountability, privacy, and cross‐border governance, especially for rare diseases where decisions are high stakes. When LLMs are used to aggregate transcriptomic evidence, map phenotypes to structured ontologies, or draft clinical reports, it may become difficult to reconstruct how a conclusion was reached, where uncertainty entered the process, or whether algorithmic error contributed to misclassification. Thus, transparency and clinical accountability are biggest obstacles for clinical translation. In rare NMD contexts, ethical issues of data privacy, informed consent, and responsible management are reported as central barriers to large‐scale health applications and international services [37]. Federated learning in rare disease is proposed to enable multi‐institutional, cross‐border collaboration without sharing raw data, improving models while preserving privacy [75]. As a result, responsible translation depends on governance models that can support collaboration without eroding patient trust, including federated learning, and clearer allocation of responsibility across developers, interpreting clinicians, healthcare institutions, and regulatory authorities.

### 5.3. Health System Implementation, Workforce Capacity, and Communication Tools

The successful implementation of multi‐omics biomarkers will depend not only on the maturity of the evidence, but also on whether health systems can absorb these tools without increasing frontline burden [76]. In rare NMDs, interpretation often requires integration of genomic findings with transcriptomic, proteomic, imaging, pathology, and longitudinal clinical data, frequently across multiple specialties. If such complexity is introduced into routine care without adequate support, multi‐omics reporting may intensify rather than relieve the workload of already stretched neurologists, geneticists, and laboratory teams [73].

For this reason, workforce readiness should be framed less as comprehensive technical retraining and more as the development of practical interpretive and governance competencies. Clinicians may not need deep expertise in every omics modality or computational method. Still, they do need to understand the strengths, limitations, confidence boundaries, and potential biases of biomarker outputs, particularly when AI‐assisted interpretation is involved [77]. In this sense, training may increasingly need to support a form of algorithmic stewardship: the ability to critically appraise computationally generated summaries, recognize when multidisciplinary review is required, and communicate uncertainty appropriately to patients and families.

Implementation of the design is equally important. Biomarker workflows should be developed around real clinical decision points rather than the assay′s technical logic alone [78]. User‐centered communication tools, structured report templates, and tiered reporting strategies may help translate high‐dimensional findings into outputs that are both clinically actionable and proportionate to user needs. For example, reports may need to distinguish clearly between core clinical conclusions, such as support for variant pathogenicity or eligibility for a trial pathway, and deeper mechanistic detail that is more appropriate for technical appendices or specialist review [77]. MDT processes may also require redesign to clearly assign responsibilities for evidence review, result verification, and downstream action [79]. Ultimately, responsible translation will depend on workflow models in which computational tools reduce data‐handling burden whereas clinicians retain responsibility for contextual interpretation and patient‐centered decision‐making.

### 5.4. Interpretation and Regulatory Pathways for AI‐Integrated Biomarker Workflows

Even when multi‐omics biomarkers show technical promise and biological plausibility, regulatory and reimbursement barriers may limit their real‐world impact. In practice, translation often depends not only on the assay itself but also on the broader diagnostic and interpretive pipeline in which it is embedded [80]. This is especially relevant when biomarker workflows include computational interpretation systems or AI‐assisted tools that support evidence synthesis, prioritization, or reporting. In such settings, regulatory oversight may need to consider both the laboratory component and the software layer, including whether parts of the workflow should be evaluated within software‐as‐a‐medical‐device frameworks and how performance should be monitored as models and pipelines evolve [81].

A related challenge is the translational “valley of death” between early biomarker promise and sustainable clinical adoption [82]. Payers and health technology assessment bodies typically require evidence not only of analytical performance, but also of incremental clinical value, downstream impact on management, and, in some systems, economic justification [83–85]. Yet in rare NMDs, generations of conventional evidence may be constrained by small populations, limited natural‐history data, heterogeneous treatment pathways, and rapidly changing standards of care [86]. Under these conditions, evidentiary expectations developed for more common diseases may not always be feasible or proportionate.

A more adaptive but accountable approach may therefore be warranted. One possible model is coverage with evidence development, in which provisional reimbursement is linked to structured collection of real‐world clinical and molecular outcomes through national or federated registries. Such approaches may help balance timely access with continued evidence generation, particularly for highly selected unsolved cases or narrowly defined clinical indications. However, regulatory flexibility should not be equated with lower standards. Rather, it should involve transparent performance thresholds, defined conditions of use, postimplementation monitoring, and ongoing reassessment of clinical value as additional data accumulate.

## 6. Future Directions

Future progress in multi‐omics translation for genetic NMDs will likely depend less on the accumulation of additional candidate signatures than on the development of more mature translational pathways. Several priorities follow from the barriers outlined above. First, future studies should place greater emphasis on fit‐for‐purpose validation, including longitudinal designs, multicenter harmonization, and clinically relevant comparators [87]. Second, biomarker development will need to be linked more explicitly to intended use, distinguishing diagnostic, stratification, prognostic, and treatment‐response applications [88]. Third, interpretive infrastructure should evolve alongside assay development. AI‐assisted tools may help reduce selected burdens in evidence synthesis, phenotype extraction, and reporting, but only within frameworks that preserve human oversight, transparency, and accountability [89]. Finally, future translation will depend on implementation strategies that are attentive to workflow, reimbursement, equity, and data governance, so that molecular advances can be evaluated not only for scientific promise, but also for their feasibility and value in routine care [90].

## 7. Conclusion

Multi‐omics biomarkers in genetic NMDs offer more than enhanced molecular resolution; especially the persistent clinical heterogeneity, variable disease trajectories, and inconsistent treatment responses. By integrating genomics, transcriptomics, proteomics, metabolomics, and epigenomics, these approaches enable improved diagnosis, patient stratification, longitudinal monitoring, and treatment‐response assessment in genetic NMDs. However, translation into routine care depends on sociotechnical readiness, including robust evidence of validity and utility, feasible workflows, equitable implementation, and appropriate governance. AI‐assisted tools may help reduce selected interpretive burdens, but they cannot replace the biological, evidentiary, and organizational foundations required for responsible translation. Thus, the central task for the field is therefore not only to identify new biomarkers, but also to actively foster multidisciplinary collaboration, prospective validation studies, and policy innovations that ensure these multi‐omics biomarkers are applicable in the real world.

## Author Contributions

Xiaoling Lang and Lunxin Liu have contributed to the work equally and should be regarded as co‐corresponding authors.

## Funding

No funding was received for this manuscript.

## Conflicts of Interest

The authors declare no conflicts of interest.

## Data Availability

Data sharing not applicable to this article as no datasets were generated or analyzed during the current study.

## References

[1] Koczwara K. E. , Lake N. J. , DeSimone A. M. , and Lek M. , Neuromuscular Disorders: Finding the Missing Genetic Diagnoses, Trends in Genetics. (2022) 38, no. 9, 956–971, 10.1016/j.tig.2022.07.001. 35908999

[2] Roque-Ramírez B. , Ríos-López K. E. , and López-Hernández L. B. , Decoding Neuromuscular Disorders: The Complex Role of Genetic and Epigenetic Regulators, Genes. (2025) 16, no. 6, 10.3390/genes16060622, 40565514. PMC12192096 40565514

[3] Cho A. , Neuromuscular Diseases: Genomics-Driven Advances, Genomics & Informatics. (2024) 22, no. 1, 10.1186/s44342-024-00027-y, 39593150. PMC11600827 39593150

[4] Thompson R. , Spendiff S. , Roos A. , Bourque P. R. , Warman Chardon J. , Kirschner J. , Horvath R. , and Lochmüller H. , Advances in the diagnosis of Inherited Neuromuscular Diseases and Implications for Therapy Development, Lancet Neurology. (2020) 19, no. 6, 522–532, 10.1016/S1474-4422(20)30028-4, 32470424. 32470424

[5] Yubero D. , Natera-de Benito D. , Pijuan J. , Armstrong J. , Martorell L. , Fernàndez G. , Maynou J. , Jou C. , Roldan M. , Ortez C. , Nascimento A. , Hoenicka J. , and Palau F. , The Increasing Impact of Translational Research in the Molecular Diagnostics of Neuromuscular Diseases, International Journal of Molecular Sciences. (2021) 22, no. 8, 10.3390/ijms22084274, 33924139. PMC8074304 33924139

[6] Hong S. E. , Kneissl J. , Cho A. , Kim M. J. , Park S. , Lee J. , Woo S. , Kim S. , Kim J. S. , Kim S. Y. , Jung S. , Kim J. , Shin J. Y. , Chae J. H. , and Choi M. , Transcriptome-Based Variant Calling and Aberrant mRNA Discovery Enhance Diagnostic Efficiency for Neuromuscular Diseases, Journal of Medical Genetics. (2022) 59, no. 11, 1075–1081, 10.1136/jmedgenet-2021-108307, 35387801. 35387801 PMC9613860

[7] Marchant R. G. , Bryen S. J. , Bahlo M. , Cairns A. , Chao K. R. , Corbett A. , Davis M. R. , Ganesh V. S. , Ghaoui R. , Jones K. J. , Kornberg A. J. , Lek M. , Liang C. , MacArthur D. , Oates E. C. , O′Donnell-Luria A. , O′Grady G. L. , Osei-Owusu I. A. , Rafehi H. , Reddel S. W. , Roxburgh R. H. , Ryan M. M. , Sandaradura S. A. , Scott L. W. , Valkanas E. , Weisburd B. , Young H. , Evesson F. J. , Waddell L. B. , and Cooper S. T. , Genome and RNA Sequencing Boost Neuromuscular Diagnoses to 62% From 34% With Exome Sequencing Alone, Annals of Clinical and Translational Neurology. (2024) 11, no. 5, 1250–1266, 10.1002/acn3.52041, 38544359. 38544359 PMC11093248

[8] McDermott H. , Sherlaw-Sturrock C. , Baptista J. , Hartles-Spencer L. , and Naik S. , Rapid Exome Sequencing in Critically Ill Children Impacts Acute and Long-Term Management of Patients and Their Families: A Retrospective Regional Evaluation, European Journal of Medical Genetics. (2022) 65, no. 9, 104571, 10.1016/j.ejmg.2022.104571, 35842091. 35842091

[9] Li J. , Zhang Y. , Jiang F. , Shi X. , Tu M. , Liu Z. , and Ye F. , Precision Medicine in Sepsis: Reappraising Glucocorticoid Therapy Through the Lens of Molecular Endotypes, Inflammation Research. (2026) 75, no. 1, 10.1007/s00011-025-02173-7, 41703348. PMC12913322 41703348

[10] Claramunt-Molet M. , Pegueroles J. , Pi-Cervera A. , Rico M. , Idelsohn-Zielonka S. , Domínguez-González C. , Corti M. , Antón V. , Salabarria S. M. , Wong K. , and James M. K. , Gait Analysis Reveals New Outcome Measures for Monitoring Disease Progression in Individuals With Late-Onset Pompe Disease, Journal of NeuroEngineering and Rehabilitation. (2026) 23, 10.1186/s12984-026-01898-8. PMC13147610 41803906

[11] Lunke S. , Bouffler S. E. , Patel C. V. , Sandaradura S. A. , Wilson M. , Pinner J. , Hunter M. F. , Barnett C. P. , Wallis M. , Kamien B. , Tan T. Y. , Freckmann M. L. , Chong B. , Phelan D. , Francis D. , Kassahn K. S. , Ha T. , Gao S. , Arts P. , Jackson M. R. , Scott H. S. , Eggers S. , Rowley S. , Boggs K. , Rakonjac A. , Brett G. R. , de Silva M. G. , Springer A. , Ward M. , Stallard K. , Simons C. , Conway T. , Halman A. , van Bergen N. J. , Sikora T. , Semcesen L. N. , Stroud D. A. , Compton A. G. , Thorburn D. R. , Bell K. M. , Sadedin S. , North K. N. , Christodoulou J. , and Stark Z. , Integrated Multi-Omics for Rapid Rare Disease Diagnosis on a National Scale, Nature Medicine. (2023) 29, no. 7, 1681–1691, 10.1038/s41591-023-02401-9, 37291213. PMC10353936 37291213

[12] Rashmi R. , Sangeetha I. K. , Sridharan K. , Keerthipriya M. S. , Vengalil S. , Atchayaram N. , Kishore Kumar R. , Sathyaprabha T. N. , and Udupa K. , Early Cardiac and Autonomic Markers and Their Genotype-Phenotype Associations in Duchenne Muscular Dystrophy, European Journal of Paediatric Neurology. (2026) 61, 47–53, 10.1016/j.ejpn.2026.03.006, 41880958. 41880958

[13] Kariyawasam D. S. , D′Silva A. , Lin C. , Ryan M. M. , and Farrar M. A. , Biomarkers and the Development of a Personalized Medicine Approach in Spinal Muscular Atrophy, Frontiers in Neurology. (2019) 10, 10.3389/fneur.2019.00898, 31481927. PMC6709682 31481927

[14] D′Silva A. , Herbert K. , Balaji L. , He J. M. , Kandula T. , Sampaio H. A. , Teoh H. L. , Tantsis E. , Sohn J. , Briggs N. , Ning N. , Kiernan M. C. , Kariyawasam D. S. , and Farrar M. A. , The Dynamics of Neurofilament Light Chain in Spinal Muscular Atrophy, Annals of Neurology. (2026) 100, no. 1, 109–122, in press 10.1002/ana.78207, 41845532. 41845532 PMC13327556

[15] Dabaj I. , Nguyen T. H. Y. , Lagrue E. , Ducatez F. , Allouche S. , Ausseil J. , Seferian A. , Gomez - Garcia de la Banda M. , Benezit A. , Phelep A. , Chouchane M. , Vasseur S. , Chapart M. , Marret S. , Quijano Roy S. , Tebani A. , and Bekri S. , Longitudinal Multi-Omics Profiling of Spinal Muscular Atrophy, Neurotherapeutics. (2026) 23, no. 2, e00880, 10.1016/j.neurot.2026.e00880, 41825231. 41825231 PMC12996660

[16] Krag T. , Nasho E. , Brady L. , Verebi C. , Leturcq F. , Malfatti E. , Duno M. , Tarnopolsky M. , and Vissing J. , Variants in CAPN3 Causing Autosomal Dominant Limb-Girdle Muscular Dystrophy Combined With Calpain-3 Deficiency, Human Mutation. (2025) 2025, 9301465, 10.1155/humu/9301465, 40226307. 40226307 PMC11972127

[17] Jones T. I. , King O. D. , Himeda C. L. , Homma S. , Chen J. C. J. , and Beermann M. L. , Individual Epigenetic Status of the Pathogenic D4Z4 Macrosatellite Correlates With Disease in Facioscapulohumeral Muscular Dystrophy, Clinical Epigenetics. (2015) 7, no. 1, 10.1186/s13148-015-0072-6, 25904990. PMC4405830 25904990

[18] Maretina M. , Koroleva V. , Shchugareva L. , Glotov A. , and Kiselev A. , The Relevance of Spinal Muscular Atrophy Biomarkers in the Treatment Era, Biomedicines. (2024) 12, no. 11, 10.3390/biomedicines12112486, 39595052. PMC11591959 39595052

[19] Ali S. S. , Li Q. , and Agrawal P. B. , Implementation of Multi-Omics in Diagnosis of Pediatric Rare Diseases, Pediatric Research. (2025) 97, no. 4, 1337–1344, 10.1038/s41390-024-03728-w, 39562738. 39562738 PMC12106075

[20] Baris S. , Ipek R. , Baris S. T. , and Baris I. , Expanding the Phenotypic Spectrum of NDUFS6-Related Disease: From Neonatal Mitochondrial Encephalopathy to Childhood-Onset Axonal Neuropathy, International Journal of Molecular Sciences. (2026) 27, no. 3, 10.3390/ijms27031375, 41683799. PMC12898639 41683799

[21] Burns J. , Timmerman V. , Laurá M. , Yiu E. M. , D’Antonio M. , Mukherjee-Clavin B. , De Winter J. , and Scherer S. S. , Charcot-Marie-Tooth Disease and Related Neuropathies, Nature Reviews Disease Primers. (2026) 12, no. 1, 10.1038/s41572-025-00679-2. 41571707

[22] Cantarero L. , Hoenicka J. , and Palau F. , Unraveling GDAP1: Bridging Mitochondrial Biology and Peripheral Neuropathy, Biomolecules. (2026) 16, no. 2, 10.3390/biom16020280, 41750352. PMC12938409 41750352

[23] Li D. , Tian L. , and Hakonarson H. , Increasing Diagnostic Yield by RNA-Sequencing in Rare Disease-Bypass Hurdles of Interpreting Intronic or Splice-Altering Variants, Annals of Translational Medicine. (2018) 6, no. 7, 10.21037/atm.2018.01.14, 29955586. PMC6015936 29955586

[24] Yang G. , Zhong H. , Wen H. , and Shen Y. , Editorial: Functional Study of Novel VUS (Variant of Uncertain Significance) Mutations in Single-Gene Inherited Disease, Frontiers in Genetics. (2025) 16, 1578736, 10.3389/fgene.2025.1578736, 40176797. 40176797 PMC11961887

[25] Roos A. , Thompson R. , Horvath R. , Lochmüller H. , and Sickmann A. , Intersection of Proteomics and Genomics to “Solve the Unsolved” in Rare Disorders Such as Neurodegenerative and Neuromuscular Diseases, PROTEOMICS–Clinical Applications. (2018) 12, no. 2, 1700073, 10.1002/prca.201700073, 29059504. 29059504

[26] Li J. , Zhu Z. , and Xu S. , A Translational Roadmap for Neurological Nonsense Mutation Disorders, International Journal of Molecular Sciences. (2026) 27, no. 3, 10.3390/ijms27031418, 41683838. PMC12897958 41683838

[27] Chassin A. , Ono H. , Ashida Y. , Imamura M. , and Aoki Y. , RNA Therapeutics for Duchenne Muscular Dystrophy: Exon Skipping, RNA Editing, and Translational Insights From Genome-Edited Microminipig Models, International Journal of Molecular Sciences. (2026) 27, no. 6, 10.3390/ijms27062755, 41898615. PMC13027266 41898615

[28] Record C. J. , Pipis M. , Skorupinska M. , Blake J. , Poh R. , Polke J. M. , Eggleton K. , Nanji T. , Zuchner S. , Cortese A. , Houlden H. , Rossor A. M. , Laura M. , and Reilly M. M. , Whole Genome Sequencing Increases the Diagnostic Rate in Charcot-Marie-Tooth Disease, Brain. (2024) 147, no. 9, 3144–3156. 38481354 10.1093/brain/awae064 PMC11370804

[29] Ueda H. , Kubota T. , Goto R. , Suzuki A. , Ojima T. , Ogawa K. , Sonehara K. , Namba S. , Naito T. , Wang Q. , Konno S. , Samukawa M. , Kawaguchi N. , Kimura T. , Sugimoto T. , Murai H. , Yamawaki T. , Kaida K. , Tokuyasu D. , Yasuda M. , Akamine H. , Onishi Y. , Ogawa Y. , Shirai Y. , Edahiro R. , Yamamoto K. , Nagata H. , Ose N. , Takayama J. , Suzuki K. , Higashiue S. , Kobayashi S. , Yamaguchi H. , Okazaki Y. , Matsumoto N. , Motomura K. , Koga H. , Japan Myasthenia Gravis Registry , BioBank Japan Project , Tohoku Medical Megabank Project Study Group , Matsuda K. , Tamiya G. , Yamamoto M. , Yamauchi T. , Kadowaki T. , Yamamoto K. , Ohno A. , Okuno T. , Morii E. , Mochizuki H. , Nagane Y. , Minami N. , Uzawa A. , Masuda M. , Suzuki S. , Shimomura I. , Shintani Y. , Utsugisawa K. , Takahashi M. P. , and Okada Y. , Elucidating Genetic Backgrounds of Myasthenia Gravis in Japanese by Genome-Wide Association Studies and Multi-Omics Analyses of Thymoma, Nature Communications. (2026) 17, no. 1, 10.1038/s41467-026-70376-5, 41820352. PMC13121629 41820352

[30] Krag T. , Nasho E. , Brady L. , Verebi C. , Leturcq F. , Malfatti E. , Duno M. , Tarnopolsky M. , and Vissing J. , Variants in CAPN3 Causing Autosomal Dominant Limb-Girdle Muscular Dystrophy Combined with Calpain-3 Deficiency, Human Mutation. (2025) 2025, no. 1, 9301465. 10.1155/humu/9301465 PMC11972127 40226307

[31] Aguti S. , Gallus G. N. , Bianchi S. , Salvatore S. , Rubegni A. , Berti G. , Formichi P. , De Stefano N. , Malandrini A. , and Lopergolo D. , Novel Biomarkers for Limb Girdle Muscular Dystrophy (LGMD), Cells. (2024) 13, no. 4, 10.3390/cells13040329, 38391941. PMC10886967 38391941

[32] Pauper M. , Hentschel A. , Tiburcy M. , Beltran S. , Ruck T. , Schara-Schmidt U. , and Roos A. , Proteomic Profiling Towards a Better Understanding of Genetic Based Muscular Diseases: The Current Picture and a Look to the Future, Biomolecules. (2025) 15, no. 1, 10.3390/biom15010130, 39858524. PMC11763865 39858524

[33] Alem L. , De Abreu Pereia D. , and Carneiro K. , The Molecular Diagnosis of Myopathies: Integrating Genomic, Proteomic, and Pathological Insights Toward Precision Medicine, Clinical Genetics. (2026) 110, no. 1, 15–28. 42089700 10.1111/cge.70180

[34] Menyhárt O. and Győrffy B. , Multi-omics approaches in cancer research with applications in tumor subtyping, prognosis, and Diagnosis, Computational and Structural Biotechnology Journal. (2021) 19, 949–960, 10.1016/j.csbj.2021.01.009, 33613862. 33613862 PMC7868685

[35] Olivier M. , Asmis R. , Hawkins G. A. , Howard T. D. , and Cox L. A. , The Need for Multi-Omics Biomarker Signatures in Precision Medicine, International Journal of Molecular Sciences. (2019) 20, no. 19, 10.3390/ijms20194781, 31561483. PMC6801754 31561483

[36] Tebani A. , Afonso C. , Marret S. , and Bekri S. , Omics-Based Strategies in Precision Medicine: Toward a Paradigm Shift in Inborn Errors of Metabolism Investigations, International Journal of Molecular Sciences. (2016) 17, no. 9, 10.3390/ijms17091555, 27649151. PMC5037827 27649151

[37] Mohr A. E. , Ortega-Santos C. P. , Whisner C. M. , Klein-Seetharaman J. , and Jasbi P. , Navigating Challenges and Opportunities in Multi-Omics Integration for Personalized Healthcare, Biomedicines. (2024) 12, no. 7, 10.3390/biomedicines12071496, 39062068. PMC11274472 39062068

[38] Vitorino R. , Transforming Clinical Research: The Power of High-Throughput Omics Integration, Proteomes. (2024) 12, no. 3, 10.3390/proteomes12030025, 39311198. PMC11417901 39311198

[39] Warman-Chardon J. , Straub V. , Vissing J. , Schlaeger S. , and Kan H. E. , 286th ENMC International Workshop: Muscle Imaging: Artificial Intelligence, Automatic Segmentation and Imaging Data Sharing in Neuromuscular Disease, Neuromuscular Disorders. (2026) 60, 10.1016/j.nmd.2025.106304. 41534227

[40] Addisu A. , Bayiyana A. , Reis Cunha J. L. , Matano D. , Younis B. M. , Hogg K. , Wiggins R. , Biwott W. , Osuna F. , Ichugu C. , Zeleke A. J. , Ayele E. , Sande J. O. , Khalil E. A. G. , Ibrahim H. M. H. , Mahmoud M. A. , Zakaria A. I. B. , Adiko B. , O’Toole P. , D’Alessio F. , Lacey C. J. N. , Mbui J. , Hailu A. M. , Kaye P. M. , Mbuchi M. , Musa A. M. , Olobo J. , and The Immstat@Cure Consortium , Systemic Inflammatory Markers of Visceral Leishmaniasis Treatment Response in East Africa, PLOS Neglected Tropical Diseases. (2026) 20, no. 2, e0013749, 10.1371/journal.pntd.0013749, 41758879. 41758879 PMC12965683

[41] Thachil A. , Wang L. , Mandal R. , Wishart D. , and Blydt-Hansen T. , An Overview of Pre-Analytical Factors Impacting Metabolomics Analyses of Blood Samples, Metabolites. (2024) 14, no. 9, 10.3390/metabo14090474, 39330481. PMC11433674 39330481

[42] Gegner H. M. , Naake T. , Dugourd A. , Müller T. , Czernilofsky F. , Kliewer G. , Jäger E. , Helm B. , Kunze-Rohrbach N. , Klingmüller U. , Hopf C. , Müller-Tidow C. , Dietrich S. , Saez-Rodriguez J. , Huber W. , Hell R. , Poschet G. , and Krijgsveld J. , Pre-Analytical Processing of Plasma and Serum Samples for Combined Proteome and Metabolome Analysis, Frontiers in Molecular Biosciences. (2022) 9, 961448, 10.3389/fmolb.2022.961448, 36605986. 36605986 PMC9808085

[43] Yu Y. , Mai Y. , Zheng Y. , and Shi L. , Assessing and Mitigating Batch Effects in Large-Scale Omics Studies, Genome Biology. (2024) 25, no. 1, 10.1186/s13059-024-03401-9, 39363244. PMC11447944 39363244

[44] Machado J. L. P. , Schaan A. P. , Mamede I. , and Fernandes G. R. , Gut Microbiota and Type 2 Diabetes Associations: A Meta-Analysis of 16S Studies and Their Methodological Challenges, Frontiers in Microbiomes. (2025) 4, 1506387, 10.3389/frmbi.2025.1506387, 41852438. 41852438 PMC12993587

[45] Tran N. L. K. , Hou X. , Heckman M. G. , Fiesel F. C. , Koga S. , Watkins M. M. , Sledge H. J. , Gibbs J. R. , Traynor B. J. , Dalgard C. L. , and Scholz S. W. , Association of Mitochondrial Genetic Background With pS65-Ub in Lewy Body Disease, Acta Neuropathologica. (2026) 151, no. 1, 10.1007/s00401-026-02993-9, 41776125. PMC12957646 41776125

[46] Kerr K. , McAneney H. , Smyth L. J. , Bailie C. , McKee S. , and McKnight A. J. , A Scoping Review and Proposed Workflow for Multi-Omic Rare Disease Research, Orphanet Journal of Rare Diseases. (2020) 15, no. 1, 107, 10.1186/s13023-020-01376-x, 32345347. 32345347 PMC7189570

[47] Osti T. , Taha A. , Reviriego-Rodrigo E. , Pastorino R. , Boccia S. , and Gutierrez-Ibarluzea I. , Value-Based Health Care Frameworks for the Health Technology Assessments of “Omics” Technologies: An International Survey, International Journal of Technology Assessment in Health Care. (2025) 41, no. 1, 10.1017/S0266462325103279, 41311259. PMC12689241 41311259

[48] Lochmüller H. , Badowska D. M. , Thompson R. , Knoers N. V. , Aartsma-Rus A. , Gut I. , Wood L. , Harmuth T. , Durudas A. , Graessner H. , and Schaefer F. , RD-Connect, NeurOmics and EURenOmics: Collaborative European Initiative for Rare Diseases, European Journal of Human Genetics. (2018) 26, no. 6, 778–785, 10.1038/s41431-018-0115-5, 29487416. 29487416 PMC5974013

[49] Rosenberg R. N. , From the Genetic Code to Neuromics, JAMA Neurology. (2013) 70, no. 6, 684–685, 10.1001/jamaneurol.2013.2854, 23571821. 23571821 PMC6768066

[50] Rath A. , Salamon V. , Peixoto S. , Hivert V. , Laville M. , Segrestin B. , Neugebauer E. A. M. , Eikermann M. , Bertele V. , Garattini S. , Wetterslev J. , Banzi R. , Jakobsen J. C. , Djurisic S. , Kubiak C. , Demotes-Mainard J. , and Gluud C. , A Systematic Literature Review of Evidence-Based Clinical Practice for Rare Diseases: What Are the Perceived and Real Barriers for Improving the Evidence and How Can They be Overcome?, Trials. (2017) 18, no. 1, 10.1186/s13063-017-2287-7, 29166947. PMC5700662 29166947

[51] Chen J. , Nie L. , Lee S. , Chu H. , Tian H. , Wang Y. , He W. , Jemielita T. , Gruber S. , Song Y. , Tamura R. , Tian L. , Zhao Y. , Chen Y. , van der Laan M. , and Lee H. , Challenges and Possible Strategies to Address Them in Rare Disease Drug Development: A Statistical Perspective, Clinical Pharmacology & Therapeutics. (2025) 118, no. 1, 62–73, 10.1002/cpt.3631, 40079686. 40079686

[52] Weymann D. , Pollard S. , Lam H. , Krebs E. , and Regier D. A. , Toward Best Practices for Economic Evaluations of Tumor-Agnostic Therapies: A Review of Current Barriers and Solutions, Value Health. (2023) 26, no. 11, 1608–1617, 10.1016/j.jval.2023.07.004, 37543205. 37543205

[53] Krebs E. , Weymann D. , Bubela T. , and Regier D. A. , How Life-Cycle Real-World Evidence Can Bridge Evidentiary Gaps in Precision Oncology, Frontiers in Medicine. (2025) 12, 1563950, 10.3389/fmed.2025.1563950, 40963574. 40963574 PMC12436484

[54] Stenzinger A. , Moltzen E. K. , Winkler E. , Molnar-Gabor F. , Malek N. , Costescu A. , Jensen B. N. , Nowak F. , Pinto C. , Ottersen O. P. , Schirmacher P. , Nordborg J. , Seufferlein T. , Fröhling S. , Edsjö A. , Garcia-Foncillas J. , Normanno N. , Lundgren B. , Friedman M. , Bolanos N. , Tatton-Brown K. , Hill S. , and Rosenquist R. , Implementation of Precision Medicine in Healthcare-A European Perspective, Journal of Internal Medicine. (2023) 294, no. 4, 437–454, 10.1111/joim.13698, 37455247. 37455247

[55] Corpas M. , Pius M. , Poburennaya M. , Guio H. , Dwek M. , Nagaraj S. , Lopez-Correa C. , Popejoy A. , and Fatumo S. , Bridging Genomics′ Greatest Challenge: The Diversity Gap, Cell Genomics. (2025) 5, no. 1, 100724, 10.1016/j.xgen.2024.100724, 39694036. 39694036 PMC11770215

[56] Portaro S. , Latella D. , Manuli A. , Calderone A. , and Calabrò R. S. , Bridging the Gap in Sexuality and Neuromuscular Disorders: A Scoping Review of an Overlooked But Crucial topic, Sexual Medicine Reviews. (2026) 14, no. 1, qeaf076, 10.1093/sxmrev/qeaf076. 41496184

[57] Llinares-Burguet I. , Sanoguera-Miralles L. , Bueno-Martínez E. , Esteban-Sanchez A. , Romano-Medina D. , Ramadane-Morchadi L. , García-Álvarez A. , Pérez-Segura P. , Easton D. F. , Devilee P. , Vreeswijk M. P. G. , de la Hoya M. , and Velasco-Sampedro E. A. , Experimental Mis-Splicing Assessment and ACMG/AMP-Guided Classification of 47 ATM Splice-Site Variants, International Journal of Molecular Sciences. (2026) 27, no. 2, 10.3390/ijms27020765, 41596415. PMC12840730 41596415

[58] Harasgama S. , Pearce H. , Appel C. , Loftus L. , Painter H. , Kuhn I. , Karpusheff J. , Ceesay A. , and Ford J. , Artificial Intelligence Tools for Automating Evidence Synthesis: Scoping Review, Journal of Medical Internet Research. (2026) 28, e81597, 10.2196/81597, 41911537. 41911537 PMC13035263

[59] Germain D. P. , Gruson D. , Malcles M. , and Garcelon N. , Applying Artificial Intelligence to Rare Diseases: A Literature Review Highlighting Lessons From Fabry Disease, Orphanet Journal of Rare Diseases. (2025) 20, no. 1, 10.1186/s13023-025-03655-x, 40247315. PMC12007257 40247315

[60] Ilić N. and Sarajlija A. , Artificial Intelligence in the Diagnosis of Pediatric Rare Diseases: From Real-World Data Toward a Personalized Medicine Approach, Journal of Personalized Medicine. (2025) 15, no. 9, 10.3390/jpm15090407, 41003110. PMC12470782 41003110

[61] Abdallah S. , Sharifa M. , Almadhoun M. K. I. K. , Khawar M. M. , Shaikh U. , Balabel K. M. , Saleh I. , Manzoor A. , Mandal A. K. , Ekomwereren O. , and Khine W. M. , The Impact of Artificial Intelligence on Optimizing Diagnosis and Treatment Plans for Rare Genetic Disorders, Cureus. (2023) 15, no. 10, e46860, 10.7759/cureus.46860, 37954711. 37954711 PMC10636514

[62] Verdu-Diaz J. , Bolano-Díaz C. , Gonzalez-Chamorro A. , Fitzsimmons S. , Warman-Chardon J. , Kocak G. S. , Mucida-Alvim D. , Smith I. C. , Vissing J. , Poulsen N. S. , Luo S. , Domínguez-González C. , Bermejo-Guerrero L. , Gomez-Andres D. , Sotoca J. , Pichiecchio A. , Nicolosi S. , Monforte M. , Brogna C. , Mercuri E. , Bevilacqua J. A. , Díaz-Jara J. , Pizarro-Galleguillos B. , Krkoska P. , Alonso-Pérez J. , Olivé M. , Niks E. H. , Kan H. E. , Lilleker J. , Roberts M. , Buchignani B. , Shin J. , Esselin F. , le Bars E. , Childs A. M. , Malfatti E. , Sarkozy A. , Perry L. , Sudhakar S. , Zanoteli E. , di Pace F. T. , Matthews E. , Attarian S. , Bendahan D. , Garibaldi M. , Fionda L. , Alonso-Jiménez A. , Carlier R. , Okhovat A. A. , Nafissi S. , Nalini A. , Vengalil S. , Hollingsworth K. , Marini-Bettolo C. , Straub V. , Tasca G. , Bacardit J. , Díaz-Manera J. , and Myo-Guide Consortium , Myo-Guide: A Machine Learning-Based Web Application for Neuromuscular Disease Diagnosis With MRI, Journal of Cachexia, Sarcopenia and Muscle. (2025) 16, no. 3, e13815, 10.1002/jcsm.13815, 40275674. 40275674 PMC12022233

[63] Scodellaro R. , Zschüntzsch J. , Hell A.-K. , and Alves F. , A first Explainable-AI-Based Workflow Integrating Forward-Forward and Backpropagation-Trained Networks of Label-Free Multiphoton Microscopy Images to Assess Human Biopsies of Rare Neuromuscular Disease, Computer Methods and Programs in Biomedicine. (2025) 265, 108733, 10.1016/j.cmpb.2025.108733, 40154003. 40154003

[64] De la Vega F. M. , Chowdhury S. , Moore B. , Frise E. , McCarthy J. , Hernandez E. J. , Wong T. , James K. , Guidugli L. , Agrawal P. B. , and Genetti C. A. , Artificial Intelligence Enables Comprehensive Genome Interpretation and Nomination of Candidate Diagnoses for Rare Genetic Diseases, Genome Medicine. (2021) 13, no. 1, 10.1186/s13073-021-00965-0, 34645491. PMC8515723 34645491

[65] Sendtner G. W. , Muecke M. , Grigull L. , Bender T. , Behning C. , and Schäfer V. S. , Cracking the Code: A Head-to-Head Comparison of Expert Clinicians and Artificial Intelligence in Diagnosing Rare Diseases, Orphanet Journal of Rare Diseases. (2025) 20, no. 1, 10.1186/s13023-025-04112-5, 41194125. PMC12590789 41194125

[66] Di Ieva A. , AI-Augmented Multidisciplinary Teams: Hype or Hope?, Lancet. (2019) 394, no. 10211, 10.1016/S0140-6736(19)32626-1. 31699402

[67] He D. , Wang R. , Xu Z. , Wang J. , Song P. , Wang H. , and Su J. , The Use of Artificial Intelligence in the Treatment of Rare Diseases: A Scoping Review, Intractable & Rare Diseases Research. (2024) 13, no. 1, 12–22, 10.5582/irdr.2023.01111, 38404730. 38404730 PMC10883845

[68] Visibelli A. , Roncaglia B. , Spiga O. , and Santucci A. , The Impact of Artificial Intelligence in the Odyssey of Rare Diseases, Biomedicines. (2023) 11, no. 3, 10.3390/biomedicines11030887, 36979866. PMC10045927 36979866

[69] Brasil S. , Pascoal C. , Francisco R. , Dos Reis F. V. , Videira P. A. , and Valadão A. G. , Artificial Intelligence (AI) in Rare Diseases: Is the Future Brighter?, Genes. (2019) 10, no. 12, 10.3390/genes10120978, 31783696. PMC6947640 31783696

[70] Grounds M. D. , Terrill J. R. , Al-Mshhdani B. A. , Duong M. N. , Radley-Crabb H. G. , and Arthur P. G. , Biomarkers for Duchenne Muscular Dystrophy: Myonecrosis, Inflammation and Oxidative Stress, Disease Models & Mechanisms. (2020) 13, no. 2, dmm043638, 10.1242/dmm.043638, 32224496. 32224496 PMC7063669

[71] Chui M. M.-C. , Kwong A. K.-Y. , Leung H. Y. C. , Pang C. , Scheller I. F. , Wong S. S.-N. , Fung C. W. , Yépez V. A. , Gagneur J. , Mak C. C. , and Chung B. H. , An Outlier Approach: Advancing Diagnosis of Neurological Diseases Through Integrating Proteomics Into Multi-Omics Guided Exome Reanalysis, Npj Genomic Medicine. (2025) 10, no. 1, 10.1038/s41525-025-00493-5, 40319040. PMC12049463 40319040

[72] Wilson L. A. , Macken W. L. , Perry L. D. , Record C. J. , Schon K. R. , Frezatti R. S. S. , Raga S. , Naidu K. , Köken Ö. Y. , Polat I. , Kapapa M. M. , Dominik N. , Efthymiou S. , Morsy H. , Nel M. , Fassad M. R. , Gao F. , Patel K. , Schoonen M. , Bisschoff M. , Vorster A. , Jonvik H. , Human R. , Lubbe E. , Nonyane M. , Vengalil S. , Nashi S. , Srivastava K. , Lemmers R. J. L. F. , Reyaz A. , Mishra R. , Töpf A. , Trainor C. I. , Steyn E. C. , Mahungu A. C. , van der Vliet P. , Ceylan A. C. , Hiz A. S. , Çavdarlı B. , Semerci Gündüz C. N. , Ceylan G. G. , Nagappa M. , Tallapaka K. B. , Govindaraj P. , van der Maarel S. , Narayanappa G. , Nandeesh B. N. , Wa Somwe S. , Bearden D. R. , Kvalsund M. P. , Ramdharry G. M. , Oktay Y. , Yiş U. , Topaloğlu H. , Sarkozy A. , Bugiardini E. , Henning F. , Wilmshurst J. M. , Heckmann J. M. , McFarland R. , Taylor R. W. , Smuts I. , van der Westhuizen F. , Sobreira C. F. D. R. , Tomaselli P. J. , Marques W Jr , Bhatia R. , Dalal A. , Srivastava M. V. P. , Yareeda S. , Nalini A. , Vishnu V. Y. , Thangaraj K. , Straub V. , Horvath R. , Chinnery P. F. , Pitceathly R. D. S. , Muntoni F. , Houlden H. , Vandrovcova J. , Reilly M. M. , and Hanna M. G. , Neuromuscular Disease Genetics in Under-Represented Populations: Increasing Data Diversity, Brain. (2023) 146, no. 12, 5098–5109, 10.1093/brain/awad254, 37516995. 37516995 PMC10690022

[73] Alemu R. , Sharew N. T. , Arsano Y. Y. , Ahmed M. , Tekola-Ayele F. , Mersha T. B. , and Amare A. T. , Multi-Omics Approaches for Understanding Gene-Environment Interactions in Noncommunicable Diseases: Techniques, Translation, and Equity Issues, Human Genomics. (2025) 19, no. 1, 10.1186/s40246-025-00718-9, 39891174. PMC11786457 39891174

[74] Smith L. A. , Cahill J. A. , Lee J.-H. , and Graim K. , Equitable Machine Learning Counteracts Ancestral Bias in Precision Medicine, Nature Communications. (2025) 16, no. 1, 10.1038/s41467-025-57216-8, 40064867. PMC11894161 40064867

[75] Süwer S. , Ullah M. S. , Probul N. , Maier A. , and Baumbach J. , Privacy-by-Design With Federated Learning Will Drive Future Rare Disease Research, Journal of Neuromuscular Diseases. (2026) 13, no. 1, 6–19, 10.1177/22143602241296276, 39973411. 39973411 PMC13141844

[76] Chen C. , Wang J. , Pan D. , Wang X. , Xu Y. , Yan J. , Wang L. , Yang X. , Yang M. , and Liu G. P. , Applications of Multi-Omics Analysis in Human Diseases, MedComm. (2023) 4, no. 4, 10.1002/mco2.315, 37533767. PMC10390758 37533767

[77] O′Connor L. M. , O′Connor B. A. , Lim S. B. , Zeng J. , and Lo C. H. , Integrative Multi-Omics and Systems Bioinformatics in Translational Neuroscience: A Data Mining Perspective, Journal of Pharmaceutical Analysis. (2023) 13, no. 8, 836–850, 10.1016/j.jpha.2023.06.011, 37719197. 37719197 PMC10499660

[78] Ivanisevic T. and Sewduth R. N. , Multi-Omics Integration for the Design of Novel Therapies and the Identification of Novel Biomarkers, Proteomes. (2023) 11, no. 4, 10.3390/proteomes11040034, 37873876. PMC10594525 37873876

[79] Reel P. S. , Reel S. , Pearson E. , Trucco E. , and Jefferson E. , Using Machine Learning Approaches for Multi-Omics Data Analysis: A Review, Biotechnology Advances. (2021) 49, 107739, 10.1016/j.biotechadv.2021.107739. 33794304

[80] Myrou A. , Barmpagiannos K. , Ioakimidou A. , and Savopoulos C. , Molecular Biomarkers in Neurological Diseases: Advances in Diagnosis and Prognosis, International Journal of Molecular Sciences. (2025) 26, no. 5, 10.3390/ijms26052231, 40076852. PMC11900390 40076852

[81] Vogeser M. and Bendt A. K. , From Research Cohorts to the Patient - A Role for “Omics” in Diagnostics and Laboratory Medicine?, Clinical Chemistry and Laboratory Medicine. (2023) 61, no. 6, 974–980, 10.1515/cclm-2022-1147, 36592431. 36592431

[82] Cho S. S. , Shin E. J. , Kim Y. G. , and Kim K. M. , Ischemic Stroke Neuroprotection Revisited: Translational Barriers and a Phase-Resolved, Biomarker-Anchored Framework, Archives of Pharmacal Research. (2026) 49, no. 1, 30–60, 10.1007/s12272-026-01593-1, 41588243. 41588243

[83] Pearson-Stuttard J. , Richardson M. , Griffin S. , Lübker C. , and Duffield S. , Closing the Gap: Do We Need a Framework for Embedding Equity in Health Technology Assessment?, International Journal of Technology Assessment in Health Care. (2026) 42, no. 1, 10.1017/S0266462325103322, 41502073. PMC12916239 41502073

[84] George P. , Eleftheria K. , and Kostas A. , Strengthening Health Technology Assessment in Greece: Industry-Identified Barriers and Recommendations, Frontiers in Public Health. (2026) 14, 1790128, 10.3389/fpubh.2026.1790128, 41883828. 41883828 PMC13010272

[85] Boss J. , Shulak L. , Rao T. , Tam C. , and Sullivan S. M. , When Should RWE Studies be Prioritized for Reimbursement? Insights From the Canadian Perspective, International Journal of Technology Assessment in Health Care. (2026) 42, no. 1, 10.1017/S0266462325103401, 41508412. PMC12964149 41508412

[86] Ferretti A. , Bianchi M. , Costa M. , Di Nardo G. , Mennini M. , Perilli L. , Perulli M. , Riva A. , Striano P. , and Parisi P. , Managing Fever and Vaccination Risks in Dravet Syndrome: From Pathophysiology to Clinical Practice, Journal of Child Neurology. (2026) 8830738261420285, 10.1177/08830738261420285, 41778674. 41778674

[87] Sabitova G. , Makhammajanov Z. , Khvan M. , Tarlykov P. , and Sazonov V. , Proteomic Biomarkers for Early Diagnosis and Prognosis in Pediatric Sepsis, World Journal of Clinical Pediatrics. (2026) 15, no. 1, 114054, 10.5409/wjcp.v15.i1.114054, 41884028. 41884028 PMC13010793

[88] Abusedera O. , Sherif J. , Karar L. , and Arekat D. , Cardiac Markers for Risk Stratification and Prognosis in Elderly Patients With HFpEF, Frontiers in Medicine. (2026) 13, 1754295, 10.3389/fmed.2026.1754295, 41788728. 41788728 PMC12956511

[89] Wang D. , Lv S. , Wei Y. , and Zhao X. , Imaging and Fluid Biomarkers for Prognostic Stratification in Cerebral Amyloid Angiopathy, Journal of Neurology. (2026) 273, no. 2, 10.1007/s00415-026-13626-2, 41559482. PMC12819511 41559482

[90] Tseng R. M. W. W. , Ong L. C. , Goh J. H. L. , Chen Y. , Chen T. , Lum E. , and Tham Y. C. , Prospective Real-World Implementation of Deep Learning Systems in Healthcare: A Systematic Review Guided by Implementation Science, NPJ Digital Medicine. (2026) 9, no. 1, 10.1038/s41746-026-02358-2, 41578007. PMC12913662 41578007

